# Foundation Engagement in Healthy Aging Initiatives and Evidence-Based Programs for Older Adults

**DOI:** 10.3389/fpubh.2014.00190

**Published:** 2015-04-27

**Authors:** Mary Ellen Kullman

**Affiliations:** ^1^Archstone Foundation, Long Beach, CA, USA

**Keywords:** evidence-based health programs, chronic disease self-management, older adults, public health and aging, Foundations, healthy aging initiatives, community health partnerships, fall prevention programs

In trying to improve health, particularly for the millions of older Americans with chronic conditions, many researchers focus solely on improving the professional health care system. Better medications, care protocols, and other clinical interventions are important, but much of chronic care takes place away from the clinic or hospital and in one’s home or community (1). Evidence-based health promotion programs (EBP) are an important way by which community agencies and health professionals can work together to offer older adults and their families proven ways to take control of their health and live the lives they want (2).

Archstone Foundation is a private independent grant making foundation with a mission of preparing society to meet the needs of an aging population. During the last two decades, the Foundation has supported the development, evaluation, and dissemination of a range of evidence-based programs in areas including fall prevention, physical activity, chronic disease management, caregiver support, and mental health. It also funded the development of Better Choices, Better Health, the online version of the Stanford Chronic Disease Self-Management Course, to broaden its reach to underserved populations. Archstone Foundation has supported the cultural adaptation of EBP to meet the needs of increasingly diverse older adults. It has funded local and national coalitions to support the dissemination of EBP, including the Falls Free Initiative led by the National Council on Aging (3). Most recently, the Foundation has funded the Evidence-Based Leadership Council to ease the challenges of dissemination and adoption of EBP by community-based organizations and health care systems.

Whether it is foundation funding, Older Americans Act funds, or resources of the organizations offering the programs, funds are limited. So are participants’ and program providers’ time and energy. The development of EBP for older adults allows limited resources to be directed to programs with the greatest probability for positive impact. As a funder looking at hundreds of requests each year, confidence in the science is critically important. We recognize that when we support any given program, other programs may struggle. EBP give funders greater assurance that what we fund will deliver meaningful results.

Archstone Foundation and many other funders have supported EBP out of a desire to improve health outcomes and quality of life for older adults. We value the programs’ self-management strategies that empower older adults, while effectively improving their health. EBP have been supported out of a desire to provide high quality, effective, and sustainable programs with a broad reach, and proven outcomes when done with fidelity to the original model (4).

Healthy aging initiatives for older adults require broad and effective community collaborations (5). Researchers, community-based organizations, older adults, health care systems, government, and funders, are all important partners in the development and effective use of EBP. Funders vary in their approach to grant making and where in the process they can engage in partnerships. Some will fund program development, while others may fund evaluation, and/or replication.

Reflecting upon the last few decades, there has been a remarkable improvement in the number, variety, and quality of evidence-based programs. A few examples of the programs include: the Chronic Disease Self-Management Program, addressing several chronic conditions; A Matter of Balance, Stepping On, and Tai Chi for Better Balance, addressing fall prevention, PEARLS (Program to Encourage Active, Rewarding Lives for Seniors), and IMPACT (Improving Mood-Promoting Access to Collaborative Treatment) addressing depression; Fit and Strong! and EnhanceFitness, addressing exercise. There is now an alternative to the free-for-all of “do it yourself” programs, developed without standards or proof of outcomes, as was the case only a few years ago. We now have a system of programs with varying levels of evidence targeting a number of health and quality of life concerns. The Centers for Disease Control and Prevention, the SAMHSA National Registry of Evidence-Based Programs and Practices, the Administration of Community Living, and the Agency for Healthcare Research and Quality are a few of the agencies that have established processes for evaluating, and certifying or registering programs as evidence-based. We are also seeing a growing number of programs addressing the needs of our diverse older adults (6). This entire Frontiers in Public Health journal issue is devoted to the study of EBP and their value.

It is important to recognize that there has been resistance to the widespread use of evidence-based programs. This resistance offers lessons in how to improve the field (7). Some have feared that EBP will stifle the creativity of practitioners and that the programs cannot respond to the unique attributes of a community, especially diverse communities. This should encourage researchers to look at how programs can be customized and better targeted to specific populations and their needs. A community-based participatory research approach that engages older adults and practitioners along with researchers in the development of new or adaptations of existing EBP may hold promise (8). Further, perceived costs and administrative barriers in offering EBP, key impediments to the spread of EBP, may suggest process improvements in these programs’ management and delivery, such as those being explored by the Evidence-Based Leadership Council. Examples of improvements to support organizations that wish to offer multiple EBP could include shared data management systems, common evaluation tools, coordinated training and technical assistance, and common core curricula (9).

Looking to the future, our challenge is to expand the breadth of offerings, improve the quality, and ease the adoption of evidence-based programs. To realize the potential of EBP, we will need to prepare our workforce to understand, implement, manage, and promote the programs (10). The use of EBP for older adults is still a relatively new phenomenon, and we are far from bringing even the most established programs to scale. Evidence will change over time, and ongoing work will be necessary. The growing diversity in the older adult population will compel us to develop, evaluate, and disseminate new EBP. There is tremendous opportunity to build partnerships and to continue to grow this exciting movement for improving health and quality of life for older adults through evidence-based programs and healthy aging initiatives.

## Conflict of Interest Statement

The author declares that the research was conducted in the absence of any commercial or financial relationships that could be construed as a potential conflict of interest.

This paper is included in the Research Topic, “Evidence-Based Programming for Older Adults.” This Research Topic received partial funding from multiple government and private organizations/agencies; however, the views, findings, and conclusions in these articles are those of the authors and do not necessarily represent the official position of these organizations/agencies. All papers published in the Research Topic received peer review from members of the Frontiers in Public Health (Public Health Education and Promotion section) panel of Review Editors. Because this Research Topic represents work closely associated with a nationwide evidence-based movement in the US, many of the authors and/or Review Editors may have worked together previously in some fashion. Review Editors were purposively selected based on their expertise with evaluation and/or evidence-based programming for older adults. Review Editors were independent of named authors on any given article published in this volume.
